# Immunoglobulin G4-related disease presenting with nephrotic syndrome due to minimal change disease: a case report

**DOI:** 10.1186/s13256-024-04494-3

**Published:** 2024-04-20

**Authors:** Amy Needleman, Michael Sheaff, Ruth J. Pepper, Rhys D. R. Evans

**Affiliations:** 1 UCL Centre for Kidney and Bladder Health, Royal Free Hospital, Pond Street, London, NW3 2QG UK; 2 Department of Histopathology, Bart’s Health NHS Trust, London, UK

**Keywords:** IgG4-related disease (IGG4-RD), IgG4-related kidney disease (IgG4-RKD), Minimal change disease (MCD), Tubulointerstitial nephritis (TIN), Necrotizing fasciitis

## Abstract

**Background:**

Immunoglobulin G4-related disease is an inflammatory disease affecting multiple organs including the kidney. Immunoglobulin G4-related kidney disease most commonly manifests as a tubulointerstitial nephritis and is associated with glomerular disease in a proportion of cases. Membranous nephropathy is the most frequent glomerular lesion. Herein, we report the first documented case of immunoglobulin G4-related disease presenting with nephrotic syndrome owing to minimal change disease.

**Case presentation:**

A 67-year-old South Asian male presented to our service with systemic upset and leg swelling. He had heavy proteinuria (urine protein:creatinine ratio 1042 mg/mmol) and was hypoalbuminemic (17 g/L) and hypercholersterolemic (9.3 mmol/L), consistent with the nephrotic syndrome. His serum creatinine was 140 μmol/L, and he was hypocomplementemic (C3 0.59 g/L, C4 < 0.02 g/L) with raised immunoglobulin G4 subclass levels (5.29 g/L). Kidney biopsy demonstrated minimal change disease alongside a plasma-cell-rich tubulointerstitial nephritis with strong positive staining for immunoglobulin G4. A diagnosis of minimal change disease in the setting of immunoglobulin G4-related disease was made. He was commenced on oral prednisolone at 60 mg daily but suffered infectious complications, including necrotizing fasciitis within 3 weeks of starting treatment, ultimately resulting in his death 52 days after initial presentation.

**Conclusion:**

This case highlights the potential for immunoglobulin G4-related disease to be associated with a spectrum of glomerular pathologies including minimal change disease. It adds to the differential diagnosis of secondary causes of minimal change disease, and moreover, aids as an important reminder of the potential complications of high-dose steroids used in its treatment.

## Background

Immunoglobulin G4-related disease (IgG4-RD) is a multisystem disease associated with lymphoplasmacytic inflammation and fibrosis. It is characterized by IgG4 + plasma cell infiltration of affected tissues and elevated serum IgG4 in some patients. Kidney involvement in IgG4-RD (IgG4-related kidney disease; IgG4-RKD) can be the result of intrinsic kidney disease or be due to ureteric obstruction from retroperitoneal fibrosis [1, 2]. Intrinsic kidney disease occurs in 12% of cases in the UK and usually consists of a tubulointerstitial nephritis (TIN) [3]. Kidney biopsy findings include lymphoplasmocytic infiltration, predominantly with IgG4+ plasma cells, with associated fibrosis that may be storiform in nature [4]. Tubular immune deposits are present in the majority of cases. TIN presents with a reduction in excretory kidney function or with mass lesions on imaging. It is associated with glomerular disease in around one-quarter of cases, which may present with edema especially if the nephrotic syndrome develops. The predominant glomerular lesion found in IgG4-RKD is membranous nephropathy (MN); IgA nephropathy and membranoproliferative glomerulonephritis have also been described [5]. Herein, we describe the first reported case of IgG4-RD presenting with minimal change disease (MCD). The case highlights the broad spectrum of glomerular pathologies associated with IgG4-RD and adds to the differential diagnosis of secondary causes of MCD.

## Case presentation

**Fig. 1:**
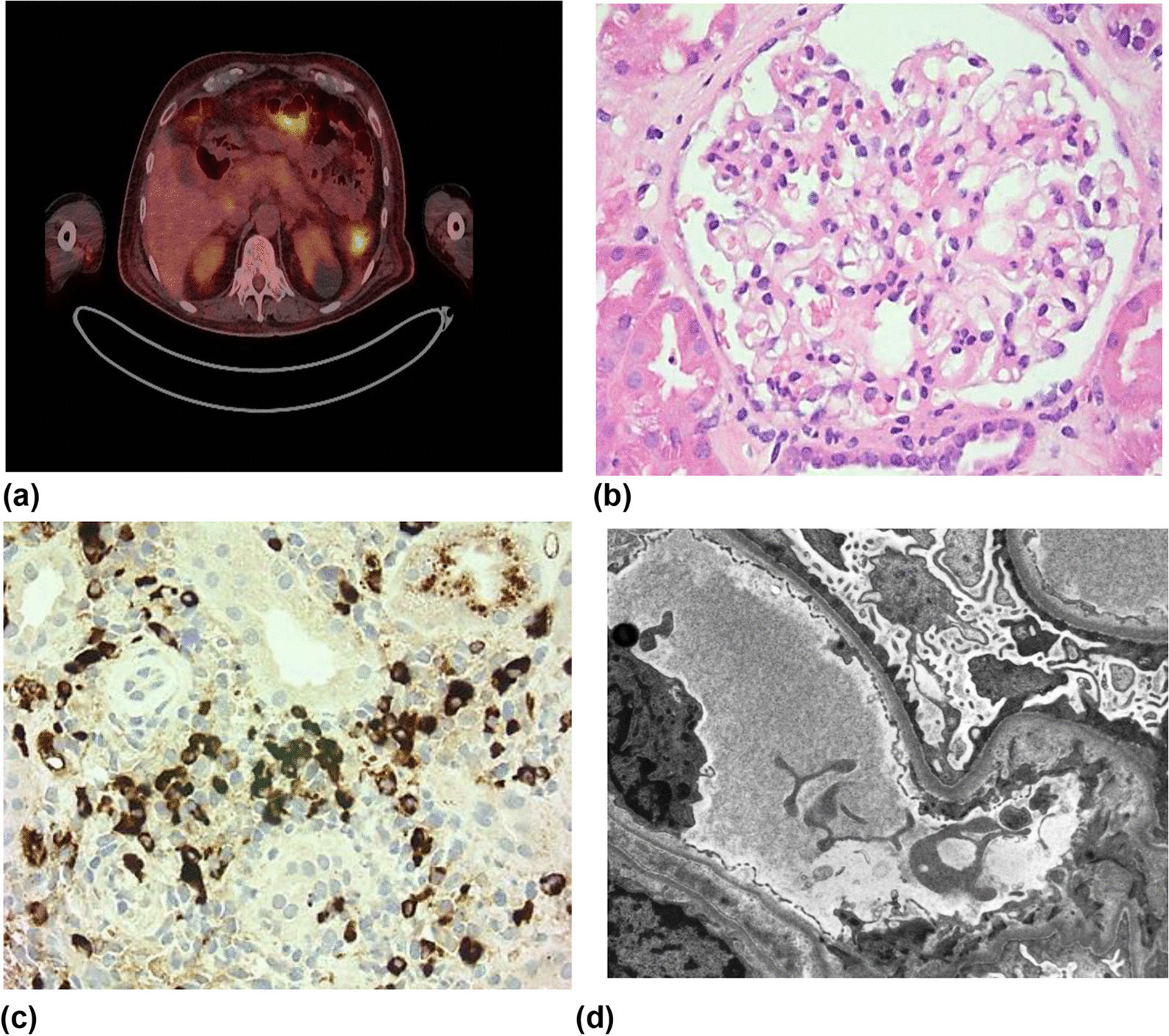
Imaging and histopathology demonstrating minimal change disease in the setting of immunoglobulin G4-related disease. **a** Positron emission tomography–computed tomography scan demonstrating splenic uptake and likely pancreatic uptake. **b** Light microscopy (hematoxylin and eosin stain; magnification × 400) demonstrating a histologically normal glomerulus. **c** Light microscopy (immunoglobulin G4 immunostain; magnification × 250) demonstrating multiple immunoglobulin G4 + plasma cells. **d** Electron microscopy demonstrating complete foot process effacement of podocytes with microvillus transformation and an absence of electron dense deposits (× 2500)

A 67-year-old male of South Asian background with history of hypertension and type 2 diabetes presented with a 4-month history of systemic upset, 7 kg weight loss, lethargy, and migratory joint pains. He was referred to our service having developed leg swelling and shortness of breath with associated significant proteinuria (urine protein:creatinine ratio 1042 mg/mmol). On examination, there was pitting peripheral edema to the mid shins without rash, embolic phenomena, or cardiac murmur. There was reduced air entry in the lungs bi-basally, blood pressure was 145/89 and oxygen saturation was 98% on room air. His serum creatinine was 140 μmol/L, and he was hypoalbuminemic (17 g/L) and hypercholersterolemic (9.3 mmol/L), consistent with the nephrotic syndrome. Further investigation demonstrated hypocomplementemia (C3 0.59 g/L, C4 < 0.02 g/L) and raised IgG4 subclass levels (5.29 g/L) (Table 1). Ultrasound showed cortical irregularity of both kidneys and a positron emission tomography-computed tomography (PET-CT) demonstrated multiple avid lymph nodes in addition to uptake in the spleen, pancreas, and prostate (Fig. 1a).

**Table 1:** Summary of patient results at presentation

Type of analysis	Investigations	Measure	Value
Urinalysis	Urine dip	Blood	2 +
		Protein	3 +
		uPCR (mg/mmol)	1042
Blood tests	Hematology	Hemoglobin (g/L)	119
		MCV (fL)	84
		White blood cell count (× 10^9^/L)	9
	Biochemistry	Creatinine (umol/L)	140
		eGFR (ml/min)	44
		Albumin (g/L)	17
		Total Cholesterol (mmol/L)	9.3
	Inflammatory markers	Crp (mg/L)	7
	Infection	Hepatitis A, B, and C serology	−ve
		HIV IgG	−ve
		EBV seroloy	Past infection
		CMV serology	Past infection
	Glomerular screen	ANCA	−ve
		Anti nuclear antibody	−ve
		Extractable nuclear antigen antibody	−ve
		Double stranded DNA	−ve
		Rheumatoid factor	−ve
		Phospholipase A2 receptor antibody	−ve
		C3 (g/L)	0.59
		C4 (g/L)	< 0.02
	Immunoglobulins	IgG (g/L)	9.6
		IgG1 (g/L)	2.94
		IgG2 (g/L)	1.74
		IgG3 (g/L)	0.85
		IgG4 (g/L)	5.29
		IgA (g/L)	1.2
		IgM (g/L)	0.5
	Myeloma screen	Protein electrophoresis	No paraprotein
		Kappa light chain (mg/L)	525.4
		Kappa:lambda light chain ratio	4.18
		Lambda light chain (mg/L)	125.6
Histopathology	Renal Biopsy	Light microscopy	**Glomeruli:** 11, 2 obsolete, remainder hypertrophic or normal
			**Tubules**: mild patchy tubular infiltrate. Focal lymphocytic tubulitis
			**Interstitium:** mild patchy scarring, plasma cells noticeable
			**Vessels:** intimal thickening of small arteries, no vasculitis
		Immunohistochemistry	Plasma cells in interstitium are IgG4 positive
		Electron microscopy	Podocyte foot process effacement suggesting MCD
Imaging	Renal USS	Kidney size (cm)	Right 12.2
		Kidney Size (cm)	Left 12.1
		Angiomyolipoma (cm)	1.5
		Simple cysts	Some
		Comments	Cortical irregularity
	PET CT	No. and location of nodes	Avid nodes above and below the diaphragm. Consistent with systemic inflammation e.g., IgG4 disease

*uPCR* urine protein creatine ratio, *MCV* mean corpuscular volume, *eGFR* estimated Glomerular Giltration rate, *CRP* C-Reactive protein, *HIV IgG* Human Immunodeficiency Virus Immunoglobulin G, *EBV* Ebstein Bar Virus, *CMV* Cytomegalovirus, *ANCA* Anti-neutrophil cytoplasmic antibody, *PET CT* Positron emission tomography–computed tomography, *MCD* minimal change disease, *Ig* immunoglobulin

The differential diagnosis for nephrotic syndrome with low (consumed) complement includes immune-complex related glomerulonephritides [such as an infection-related glomerulonephritis (GN), membranoproliferative GN, lupus nephritis, and Sjögren’s syndrome-associated GNs], cholesterol embolization, and glomerular disease in the setting of IgG4-RD. The patient underwent diagnostic kidney biopsy to differentiate between these potential causes. Glomeruli were unremarkable on light microscopy with no glomerular immunoglobulin staining (Fig. 1b). Electron microscopy demonstrated complete foot process effacement without the presence of electron dense deposits, consistent with MCD (Fig. 1c). In addition, there was a plasma-cell-rich interstitial infiltrate with strong positive staining for IgG4 (52 IgG+ plasma cells/high power field and 60% IgG4/IgG ratio), fulfilling Raissan’s diagnostic criteria for IgG4-RKD [6] (Fig. 1d). A unifying diagnosis of MCD in the setting of IgG4-RKD was made.

The patient was treated with oral prednisolone at 60 mg daily alongside conservative measures for the nephrotic syndrome (angiotensin converting enzyme inhibition, furosemide, atorvastatin, and heparin). There was no significant glomerular response, and on day 17 he presented to hospital with an enlarging erythematous, macular rash on his thigh, which was extremely tender to palpation. He was systemically unwell with fever and a clinical diagnosis of necrotizing fasciitis was made. Despite intravenous antibiotics and multiple surgical debridements, he became increasingly septic requiring ventilatory and renal support within intensive care. Immunosuppression was reduced, and intravenous immunoglobulin was given owing to new-onset hypogammaglobulinemia. Despite this, he developed bacteremia (*Enterobacter cloacae* and *Enterococcus faecium*) and fungemia (*Candida albicans*) with resultant mitral valve endocarditis. His disease course culminated in acute bowel ischemia and a decision was made at this point to provide palliative care. The patient died 52 days after initial presentation.

## Discussion

Glomerular diseases, including those that result in the nephrotic syndrome, may be primary processes or secondary to systemic disease and medications. MCD in both children and adults is most often primary, thought to be the result of T cell dysfunction and the production of a circulating permeability factor. Secondary causes, however, should be specifically excluded as their presence may impact management decisions, including targeting of the precipitating factor or disease process. Secondary causes of MCD include medications (such as nonsteroidal anti-inflammatory drugs), malignancies (most commonly lymphoproliferative diseases), infections, and allergy [7]. While MCD has previously been reported in Japan in a patient with known IgG4-RD [8], to the best of our knowledge, this is the first reported case of IgG4-RD presenting with MCD and the first case of IgG4-related MCD in a patient in Europe.

IgG4-RKD is associated with diverse renal manifestations. It was initially reported in cohorts predominantly from Asia and North America [9–12], but more recent studies are reported from Europe [3, 13]. Intrinsic kidney disease occurs in 7–44% of patients and TIN is the most common histological lesion. This may be associated with glomerular disease in 9–39% of cases. We previously demonstrated that, in a UK-based cohort, 27% of TIN cases had glomerular disease; all glomerular disease in this cohort was MN [3]. This included one case of MN with no associated TIN, highlighting the potential for glomerular disease in IgG4-RD in isolation without tubulointerstitial inflammation. The pathogenesis of glomerular disease in IgG4-RD is unclear, irrespective of the pattern of glomerular injury. Patients with MN do not have detectable autoantibodies to the phospholipase A2 receptor, although antibodies to other podocyte antigens have been described [14]. The absence of immune deposits in MCD goes against this being an antibody mediated process; whether T cell dysfunction (as part of wider dysfunction of the immune system) or a circulating factor contributes to the pathogenesis of MCD in IgG4-RD are unexplored. There are no randomized controlled studies to guide the management of IgG4-RKD. TIN is often responsive to steroid therapy, but frequently relapses and steroid sparing agents are increasingly used. There are even less data to guide the management of IgG4-related glomerular disease. We approach management as we would for the respective primary glomerular lesion and aim to make management decisions within a dedicated IgG4-RD multidisciplinary team.

## Conclusion

This case demonstrates both the need to consider IgG4-RD as a secondary cause of MCD (particularly in patients with hypocomplementemia which is not associated with primary MCD) and supports an increasing awareness of the broad range of glomerular pathologies associated with IgG4-RKD. It highlights the need for clinicians to be aware of IgG4-RKD as a potential cause in patients presenting with the nephrotic syndrome. Moreover, it reinforces the need for further research into the pathogenesis of glomerular disease in IgG4-RD, in addition to the need for more data to guide its management.

## Data Availability

The datasets during and/or analyzed during the current study are available from the corresponding author upon reasonable request.
